# A daily glass of red wine associated with lifestyle changes independently improves blood lipids in patients with carotid arteriosclerosis: results from a randomized controlled trial

**DOI:** 10.1186/1475-2891-12-147

**Published:** 2013-11-15

**Authors:** Dirk W Droste, Catalina Iliescu, Michel Vaillant, Manon Gantenbein, Nancy De Bremaeker, Charlotte Lieunard, Telma Velez, Michèle Meyer, Tessy Guth, Andrea Kuemmerle, Georges Gilson, Anna Chioti

**Affiliations:** 1 Department of Neurology, Centre Hospitalier de Luxembourg (CHL), 4 rue Barblé, L-1210, Luxembourg, Luxemburg; 2 Centre de Recherche Public-Santé (CRP-Santé), Clinical and Epidemiological Investigation Centre (CIEC), 1A-B, rue Thomas Edison, L-1445, Strassen, Luxemburg; 3 Centre de Recherche Public-Santé (CRP-Santé), Methodology and Statistical Competence Centre (CCMS), 1A-B, rue Thomas Edison, L-1445, Strassen, Luxemburg; 4 Centre de Recherche Public-Santé (CRP-Santé), Centre of Health Studies, 1A-B, rue Thomas Edison, L-1445, Strassen, Luxemburg; 5 Department of Clinical Biology, Centre Hospitalier de Luxembourg (CHL), 4 rue Barblé, L-1210 Luxemburg, Luxembourg

**Keywords:** Alcohol, Carotid arteries, Diet, Lipids, Nutrition

## Abstract

**Background:**

Physical exercise and a Mediterranean diet improve serum lipid profile. The present work studied whether red wine has an effect on top of a lipid-lowering lifestyle in patients with carotid atherosclerosis.

**Methods:**

A prospective randomised unblinded trial was performed from 2009 to 2011 in 108 patients with carotid atherosclerosis, 65% of whom were already on statin therapy with a low mean LDL of 104.9 mg/dl. Half of them were advised to follow a modified Mediterranean diet and to perform moderate physical exercise during 30 min/day (lifestyle changes) for 20 weeks. Within these two groups half of the patients were randomised either to avoid any alcohol or to drink 100 ml of red wine (women) or 200 ml of red wine (men) daily.

**Results:**

LDL was significantly lowered by 7% in the lifestyle-changes group compared to the no-lifestyle-changes group (p = 0.0296) after 20 weeks. Lifestyle changes lowered the LDL/HDL ratio after 20 weeks by 8% (p = 0.0242) and red wine independently by 13% (p = 0.0049). The effect on LDL/HDL ratio after 20 weeks was, however, more pronounced in the non-LC group. Total cholesterol (−6%; p = 0.0238) and triglycerides (−13%; p = 0.0361) were lowered significantly by lifestyle changes after 20 weeks compared to the no-lifestyle-changes group. Lipoprotein (a) was not significantly affected by any intervention. The given results are per ITT analysis.

**Conclusions:**

Lifestyle changes including a modified Mediterranean diet and physical exercise as well as a glass of red wine daily improve independently the LDL/HDL ratio in patients with carotid arteriosclerosis even though the vast majority of them was already on statin therapy.

**Trial registration:**

http://www.clinicaltrials.gov, NCT01146132

## Background

There is a huge body of evidence from observational and interventional studies that high serum total cholesterol, LDL cholesterol and lipoprotein(a) (Lp(a)) levels, as well as low HDL-cholesterol levels are related to cerebral and cardiac vascular diseases [1-8]. The role of triglycerides as an independent risk factor is less consistent as their level clusters with other risk factors [2,9,10].

Lifestyle changes (LC) including physical exercise and a Mediterranean diet improve lipid profile. Physical exercise mainly increases HDL and reduces triglycerides; LDL could be affected [11,12]. In an interventional trial a Mediterranean diet enriched with mixed nuts or extra-virgin olive oil decreased LDL cholesterol and triglycerides, and increased HDL cholesterol [13]. In their recent meta-analysis, Kastorini et al. found an increase in HDL and a decrease in triglycerides when comparing a Mediterranean diet with conventional diet [14]. Some of the studies in this meta-analysis included red wine, others not. Three food items in particular have been shown to improve lipid profile: dark chocolate, tomatoes and walnuts, the latter two being frequently part of a Mediterranean diet [15-20]. Light to moderate alcohol consumption (up to 1 drink daily for women and 1 or 2 drinks daily for men) and possibly in particular red wine consumption is associated with less cerebro- and cardiovascular diseases and an improved lipid profile [21-26]. Regarding red wine consumption (and other food items) the question is still open whether lifestyle associated with moderate red wine consumption or wine itself cause less cerebro-cardiovascular disease [27]. Only in February 2013 the results of a large prospective randomised trial were published showing a benefit of mediterranean diet namely on stroke risk in comparison to a low-fat diet (relative risk reduction of 33%-46%) [28].

In the present prospective unblinded randomised controlled trial we assessed the effect of a small amount of red wine and LC including the consumption of dark chocolate, tomatoes and walnuts associated with physical activity advice on lipid profile in patients with arteriosclerosis documented by carotid ultrasound. In particular we studied whether the use of red wine on top of LC still results in an additional lipid profile improvement. The present work is part of the ALVINA project (an acronym of “**al**imentation, **vin** et **a**ctivité physique” which mean “nutrition, wine, and physical activity” in French), where also other parameters were studied.

## Methods

### Population analysed

122 patients were followed up to 20 weeks. The enrolled participants were out-patients of the Neurology department of the Centre Hospitalier de Luxembourg and had undergone carotid and intracranial bitemporal color coded duplex sonography using an Antares system (Siemens Healthcare, 91052 Erlangen, Germany). Inclusion criteria were >30 years and the presence of plaques or stenosis without hemodynamic compromise (i.e. <70%) in at least one common carotid artery, the carotid bifurcation, or the internal carotid artery. We used the Mannheim definition for plaques: It is defined as a focal structure that encroaches into the arterial lumen of at least 0.5 mm or 50% of the surrounding IMT value or demonstrates a thickness >1.5 mm as measured from the media-adventitia interface to the intima-lumen interface [29]. NASCET criteria were used to define high-grade stenosis [30,31]. Exclusion criteria were a history of ocular or cerebral ischemia within the last 3 months, atrial fibrillation, or the incapacity to give informed consent.

This study was conducted according to the guidelines laid down in the Declaration of Helsinki and all procedures involving human subjects/patients were approved by the national research ethics committee (200801/06) and notified to the national commission on data protection in Luxemburg. Written informed consent was obtained from all subjects. The trial was registered (http://www.clinicaltrials.gov, NCT01146132). The first patient was included on June 4th 2009 and the last follow-up visit was on October 10th 2011. The study was performed in Luxemburg.

### Intervention administered

The design of the study was a 2 × 2 factorial design. The patients were attributed to groups of lifestyle in result of a randomization stratified for gender. Lots were drawn to generate the random allocation sequence. Block size was four. The intervention allocation was concealed in a non-transparent envelope. Random allocation sequence was generated by an independent staff member; patients were enrolled by the principal investigator; patients were assigned to interventions by study nurse. The first group received no lifestyle counseling and the second one received lifestyle counseling at the beginning, after 1 week, after 2 weeks, after 3 weeks and after 4 weeks. Within each lifestyle group patients were either randomly advised to drink a glass of red wine a day (0.2 l for men and 0.1 l for women), or advised to avoid alcohol at all for the time of the study. The sort of red wine was at the participants’ discretion.

**Figure 1:**
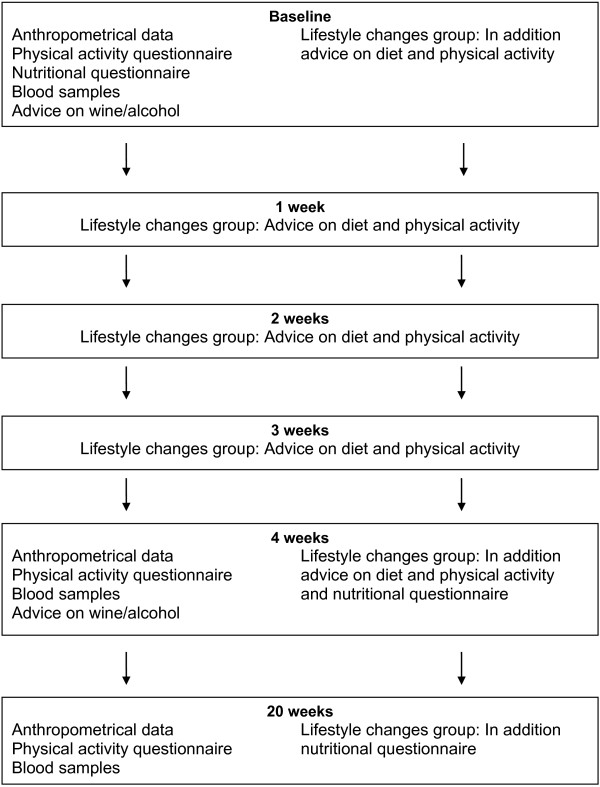
Study procedures.

Figure 1 gives an overview of the study procedures. Only in the LC group, a dietician performed five sessions of 30 min each (baseline, after one week, after 2 weeks, after 3 weeks and after 4 weeks) giving advice on healthy eating based on a modified Mediterranean diet and physical exercise. In particular, 5 portions of fruit/vegetables per day, a diet low in absolute fat, a preference of vegetable oil (olive or canola oil), whole grain products, poultry, low fat dairy products, a fat and a lean fish meal per week, a reduced consumption of red meat, and the avoidance of pork, of ready-made meals, of sugar and of excessive salt intake were recommended [32,33]. A water intake of 1.5-2 l a day was recommended as well. In addition the regular consumption of one bar of dark chocolate (25 g, >70% of cacao), 1–2 tomatoes, and of 3–5 walnuts as well as at least 30 min of moderate daily physical activity were recommended [32,34,35]. In the literature the quantities of dark chocolate, tomatoes, and walnuts are higher [17,20,36,37]; however we used quantities applicable in daily life over a long period. The control group (no LC) was seen at baseline, after 4 weeks and after 20 weeks by a nurse and instructed not to change their eating and physical activity habits. The patients allocated to the wine group (within the LC and the no-LC groups) underwent three short sessions of counseling concerning the intake of red wine of their choice (0.2 l for men and 0.1 l for women) by the nurse [24,38,39].

### Analyses performed

All participants were initially seen by the dietician for a baseline assessment of their nutrition habits over the last 2 working days and the last weekend day using the software Nutrilog Pro (Nutrilog SAS, Marans, France). The participants in the lifestyle-changes group were reassessed at 4 weeks and at 20 weeks using the same method. General anthropometrical data such as weight, height, body-mass index were recorded. Physical activity over the week preceding the clinical visit was assessed using a questionnaire. Physical activity was differentiated into light (no increase in respiration and sweating), moderate (increase in respiration, no sweating) and intense physical activity (increased respiration and sweating) [32,34,35]. Daily minutes of each exercise level were calculated. A scale (model 763 1321009, Seca, Hamburg, Germany) was used to assess body weight and a meter to assess height. Fasting total cholesterol, LDL and HDL cholesterol, triglycerides, and Lp(a) at baseline, after 4 weeks and 20 weeks were measured from serum samples collected after a 12 hour fast. Total cholesterol, HDL-cholesterol, LDL-cholesterol, triglycerides and Lp(a) were assessed on a Modular P module (Roche, Basel, Switzerland). Total cholesterol, HDL-cholesterol, LDL-cholesterol and triglycerides were all determined by enzymatic colorimetric assays (all Roche, Basel, Switzerland). An immunoturbidimetric assay (Roche, Basel, Switzerland) was used to measure Lp(a).

### Statistical analysis

The pre-defined primary endpoint of the study was a change in the ratio LDL/HDL cholesterol assessed at baseline and at 20 weeks. Secondary endpoints were changes in the other above-mentioned blood parameters and body weight/BMI and the above mentioned changes after 4 weeks. Extrapolated data from previous studies allowed calculation of LDL/HDL ratio change between baseline and after diet or wine intake [25,40,41]. The extractions from the articles resulted in a 0.03 change in the no diet group with no wine, 0.1 in the diet group with no wine and the no diet group with wine, 0.48 in the diet group with wine. The standard deviation was extrapolated to 0.02. Assuming an alpha risk of 0.025 (Bonferroni corrected for two comparisons) and a power of 0.9, the total sample size should be 88 to show a significant difference between groups by using a general linear model including the diet and wine groups as well as their interaction. Allowing for 15% of loss to follow-up, the sample size should finally be 100 patients.

The intent-to-treat analysis (ITT) included only patients who had attended the first and at least the 4 weeks visit. The per-protocol-analysis (PP) included ITT patients and excluded those who admitted not to have followed the instructions or who did not present themselves at the 20-week visit. The ITT population was the primary population. The groups (defined by LC and red wine intake) were compared for their baseline values. For continuous variables, normality was verified using the Shapiro-Wilk test. Where normally distributed, a two-sample t-test was used. Otherwise the Wilcoxon-Mann–Whitney test was applied. Dichotomous data was compared using a Pearson’s chi-squared test. In case expected cell frequencies were lower than 5, Fisher’s exact test was applied. A general linear model with Tukey-Kramer adjustment for multiple comparisons was used to assess the primary endpoints. The change value represented the dependent value. Lifestyle group (conventional, LC), wine group (with wine, without wine) and the interaction between lifestyle and wine group were included in the model as independent variables as well as the baseline value as a covariate. In case where the normality assumption of the model residuals was violated, a non-parametric van-Elteren test was used with stratification for the other intervention (i.e. when comparing LC vs. no LC, stratification for wine group was used and vice versa). We looked for an imbalance in statin treatment between the groups by the chi-square test at baseline, after 4 weeks, and after 20 weeks. A p-value below 0.05 was considered significant. All tests were two-tailed. All analyses were performed by using the SAS System v9.2 (SAS Institute, Cary, NC, USA).

## Results

**Figure 2:**
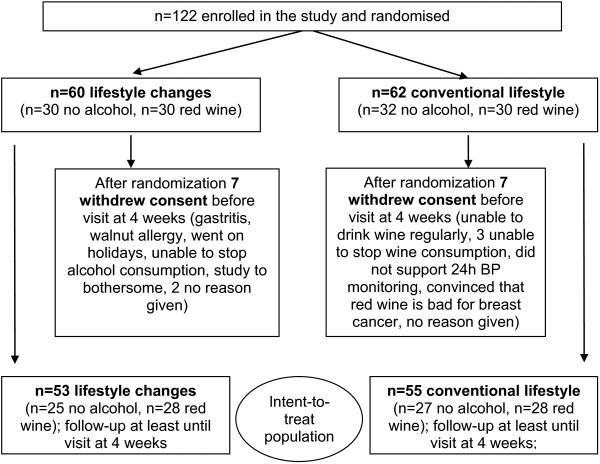
Patient attrition.

108 patients, 36 women and 72 men aged from 37 to 83 years (mean 64±9 years) were included in the ITT population (cf. Figure 2).

Among all baseline parameters (demographic, medical history, lipid profile, nutritional values and physical activity), no difference was found between groups (cf. Tables 1 and 2). There was homogeneity between the groups except for the fact that at baseline, subjects in the no-red-wine group consumed more lipids, proteins and mono-unsaturated fatty acids and less red wine than subjects in the red-wine group. No serious adverse event related to the intervention (LC or wine) was recorded.

**Table 1:** Baseline demographic values, past medical history, treatment, and lipid profile (SD in brackets)

	**No LC (n = 55)**	**LC (n = 53)**	**p-value**	**Red wine (n = 56)**	**No red wine (n = 52)**	**p-value**	**Total**
Mean age [years]	63.4 (10.6)	63.7 (8.1)	NS	64.1 (9.1)	63.0 (9.9)	NS	63.6 (9.5)
Men [%]	69	64	NS	68	65	NS	67
Mean weight [kg]	81.6 (16.2)	77.2 (16.3)	NS	79.5 (14.3)	79.4 (18.4)	NS	79.4 (16.3)
Mean BMI [kg/m^2^]	27.8 (4.2)	27.3 (4.5)	NS	27.4 (3.9)	27.7 (4.8)	NS*	27.6 (4.4)
Smoker [%]	11	4	NS	5	10	NS	7
Hypertension [%]	69	66	NS	71	63	NS	68
Hyper-/Dyslipidemia [%]	76	72	NS	71	77	NS	74
Diabetes mellitus [%]	13	11	NS	14	10	NS	12
Previous stroke [%]	20	21	NS	25	15	NS	20
Previous TIA [%]	15	9	NS	11	14	NS	12
Previous MI [%]	11	8	NS	7	12	NS	9
Angina pectoris [%]	7	4	NS	5	6	NS	6
Intermittent claudication [%]	0	2	NS	0	2	NS	1
On statin [%]	65	64	NS	61	69	NS	66
On fibrate [%]	2	4	not tested	4	2	not tested	3
On nicotinic acid [%]	0	2	not tested	2	0	not tested	1
On ezetrol [%]	9	2	not tested	4	8	not tested	6
On fish oil supplements	2	6	not tested	2	6	not tested	4
Mean total cholesterol [mg/dL]	178.5 (4.9)	176.2 (5.4)	NS	178.0 (5.2)	176.8 (5.1)	NS	177.4 (37.4)
Mean total cholesterol [mmol/L]	4.62 (0.13)	4.56 (0.14)	NS	4.60 (0.13)	4.57 (0.13)	NS	4.59 (0.97)
Mean LDL [mg/dL]	105.0 (32.7)	104.8 (32.6)	NS*	106.5 (33.3)	103.2 (31.8)	NS*	104.9 (32.5)
Mean LDL [mmol/L]	2.72 (0.85)	2.71 (0.84)	NS*	2.75 (0.86)	2.67 (0.82)	NS*	2.71 (0.84)
Mean HDL [mg/dL]	59.1 (19.6)	57.2 (14.3)	NS*	55.9 (15.3)	60.7 (18.8)	NS*	58.2 (17.2)
Mean HDL [mmol/L]	1.53 (0.51)	1.48 (0.37)	NS*	1.45 (0.40)	1.57 (0.49)	NS*	1.51 (0.44)
LDL/HDL	2.0 (0.8)	1.9 (0.7)	NS*	2.0 (0.8)	1.8 (0.8)	NS*	1.9 (0.8)
Mean triglycerides [mg/dL]	109.1 (77.5)	99.7 (42.0)	NS*	115.7 (78.0)	92.7 (38.1)	NS*	104.5 (62.7)
Mean triglycerides [mmol/L]	1.23 (0.88)	1.13 (0.47)	NS*	1.31 (0.88)	1.05 (0.43)	NS*	1.18 (0.71)
Mean Lp(a) [mg/dL]	56.9 (48.8)	56.1 (64.4)	NS*	68.2 (67.9)	45.3 (41.8 )	NS*	56.5 (57.0)
Mean Lp(a) [mmol/L]	2.03 (1.74)	2.00 (2.30)	NS*	2.43 (2.42)	1.62 (1.49)	NS*	2.02 (2.03)

No differences were found between the LC and the no LC group, as well as between the red wine and the no red wine group in Student’s t tests, Wilcoxon-Mann–Whitney tests (indicated by *). Categorical values were tested with the Chi-square test or Fisher’s exact test.

**Table 2:** Baseline daily nutritional values and physical activity based on ITT population (mean, SD in brackets)

	**No LC (n = 55)**	**LC (n = 53)**	**LC effect**	**Red wine (n = 56)**	**No red wine (n = 52)**	**Wine effect**	**Total**
Total caloric intake [kJ]	8113 (1928)	8222 (2072)	NS	7841 (1946)	8523 (2000)	NS	8167 (1992)
Total caloric intake [kcal]	1939 (460.8)	1965 (495.3)	NS	1874 (465.1)	2037 (477.9)	NS	1952 (476.1)
Carbohydrates [g]	221.8 (62.5)	218.4 (68.8)	NS*	214.9 (68.2)	225.8 (62.4)	NS*	220.1 (65.4)
Lipids [g]	69.8 (21.9)	72.8 (25.4)	NS	66.1 (22.2)	77.0 (24.1)	0.0162	71.3 (23.6)
Proteins [g]	78.8 (17.0)	82.6 (20.5)	NS*	77.0 (16.6)	84.8 (20.4)	0.0304	80.7 (18.9)
Vegetables [g]	244.2 (145.3)	248.9 (138.4)	NS*	237.5 (116.5)	256.5 (165.0)	NS*	246.5 (141.3)
Fruits [g]	237.6 (203.3)	235.9 (208.0)	NS*	249.2 (202.2)	223.0 (208.5)	NS*	236.7 (204.7)
Fibres [g]	20.2 (8.2)	18.8 (8.2)	NS*	20.7 (8.0)	18.1 (8.2)	NS*	19.5 (8.2)
Mono-unsaturated fatty acids [g]	21.2 (7.9)	22.7 (8.8)	NS*	20.5 (8.2)	23.6 (8.3)	0.0317*	22.0 (8.3)
Poly-unsaturated fatty acids [g]	9.4 (3.5)	10.2 (5.2)	NS*	9.2 (3.5)	10.5 (5.2)	NS*	9.8 (4.4)
Walnuts [number]	0.5 (1.0)	0.4 (1.5)	NS*	0.4 (0.9)	0.4 (1.6)	NS*	0.4 (1.2)
Tomatoes [number]	0.4 (0.4)	0.3 (0.5)	NS*	0.3 (0.4)	0.4 (0.5)	NS*	0.3 (0.5)
Dark chocolate [g]	3.8 (6.9)	3.6 (9.8)	NS*	4.5 (10.3)	2.9 (5.8)	NS*	3.7 (8.4)
Total alcohol [g]	14.7 (14.7)	14.5 (14.2)	NS*	15.4 (14.3)	13.7 (14.6)	NS*	14.6 (14.4)
Red wine [mL]	69.8 (98.3)	99.5 (114.7)	NS*	102.4 (107.4)	64.8 (104.6)	0.0238*	84.5 (107.2)
White wine [mL]	46.9 (70.6)	26.9 (51.6)	NS*	30.7 (59.2)	43.8 (65.8)	NS*	37.0 (62.5)
Rosé wine [mL]	9.6 (49.3)	1.9 (13.7)	NS*	4.5 (33.4)	7.2 (39.6)	NS*	5.8 (36.3)
Beer [mL]	56.6 (133.8)	39.5 (117.9)	NS*	36.9 (104.1)	60.4 (146.1)	NS*	48.1 (125.8)
Other alcohols [mL]	2.0 (5.9)	2.4 (8.2)	NS*	2.8 (7.6)	1.6 (6.5)	NS*	2.2 (7.1)
Mild physical activity [min]	282.1 (222.1)	244.6 (180.3)	NS*	253.1 (199.8)	275.2 (206.9)	NS*	263.7 (202.6)
Moderate physical activity [min]	54.2 (93.4)	44.4 (72.1)	NS*	44.0 (62.8)	55.2 (101.4)	NS*	49.4 (83.4)
Intensive physical activity [min]	25.7 (60.5)	17.3 (31.3)	NS*	20.6 (50.9)	22.7 (46.1)	NS*	21.6 (48.4)

P-values are from homogeneity tests (Student’s t-test and Wilcoxon-Mann–Whitney test indicated by *, respectively).

The results for the primary endpoint and the secondary endpoints are presented in Table 3. Four weeks after beginning the intervention, LC and no-LC groups showed no significant differences in blood lipids. Significant differences were observable for HDL cholesterol (p = 0.0263) and LDL/HDL ratio (p = 0.0293). After 20 weeks, LDL was significantly lowered by 7% in the lifestyle-changes group compared to the no-lifestyle-changes group (p = 0.0296). LC lowered the LDL/HDL ratio after 20 weeks by 8% (p = 0.0242) and red wine independently by 13% (p = 0.0049). Total cholesterol (−6%; p = 0.0238) and triglycerides (−13.2%; p = 0.0361) were lowered significantly by LC after 20 weeks compared to the no-LC group. Interaction between LC and wine was not significant in any analysis of the outcomes with the general linear model. Consequently LC and wine can be considered as independent factors. Weight and BMI did not change after 20 weeks; there was only a short effect after 4 weeks in the LC group (0.7%). Lp (a) was not affected by any intervention. No significant imbalance was found in statin use throughout the study. Results obtained on the PP population were similar.

**Table 3:** Change of blood lipids and weight during the study (in%, SD in brackets)

	**No LC (n = 55)**	**LC (n = 53)**	**p for LC effect**	**Red wine (n = 56)**	**No red wine (n = 52)**	**p for red-wine effect**	**No LC, no wine (n = 27)**	**No LC, red wine (n = 28)**	**LC, no wine (n = 25)**	**LC, red wine (n = 28)**
**Baseline - 4 weeks**										
Total cholesterol	0.9 (2.3)	−4.2 (1.8)	NS	−0.6 (2.1)	−2.6 (2.1)	NS	0.4 (17.0)	1.4 (16.4)	−6.0 (12.4)	−2.6 (13.9)
LDL	1.5 (20.4)	−5.3 (17.6)	NS*	−2.5 (20.3)	−1.1 (18.4)	NS*	2.4 (19.3)	0.6 (21.8)	−5.1 (16.9)	−5.5 (18.5)
HDL	1.1 (2.2)	−2.1 (1.5)	NS	2.8 (1.8)	−3.8 (1.9)	0.0263	−2.8 (16.8)	5.0 (15.0)	−5.1 (9.4)	0.6 (11.4)
LDL/HDL	1.7 (20.9)	−2.6 (18.7)	NS*	−4.5 (18.9)	3.9 (20.2)	0.0293*	6.9 (20.9)	−3.5 (19.9)	0.6 (19.2)	−5.4 (18.2)
Triglycerides	1.1 (29.3)	−1.2 (30.3)	NS*	−2.0 (29.2)	2.1 (30.4)	NS*	4.3 (29.6)	−2.1 (29.2)	−0.4 (31.6)	−1.9 (29.7)
Lp (a)	1.6 (21.3)	9.0 (32.7)	NS*	5.6 (25.7)	4.9 (29.7)	NS*	0.8 (23.9)	2.5 (18.7)	9.4 (35.1)	8.5 (31.1)
Weight/BMI	0.0 (1.4)	−0.7 (1.8)	0.0253*	−0.1 (1.5)	−0.6 (1.8)	NS*	−0.4 (1.4)	0.4 (1.3)	−0.8 (2.1)	−0.7 (1.5)
**Baseline - 20 weeks**										
Total cholesterol	3.6 (2.2)	−2.4 (2.0)	0.0238	−0.9 (2.0)	2.4 (2.3)	NS	7.2 (17.6)	0.5 (13.8)	−2.5 (13.0)	−2.3 (15.4)
LDL	4.4 (23.3)	−3.2 (19.1)	0.0296*	−3.3 (20.5)	5.1 (22.1)	NS*	11.7 (25.2)	−2.0 (20.0)	−1.5 (16.4)	−4.6 (21.4)
HDL	2.1 (1.9)	2.0 (1.9)	NS	4.2 (1.9)	−0.3 (1.9)	NS	−1.5 (12.9)	5.3 (14.4)	0.9 (13.5)	3.0 (13.1)
LDL/HDL	4.3 (28.0)	−4.1 (20.6)	0.0242*	−6.1 (20.8)	7.3 (27.2)	0.0049*	15.7 (30.8)	−5.5 (21.3)	−1.1 (20.5)	−6.7 (20.7)
Triglycerides	7.0 (35.7)	−6.2 (34.7)	0.0361*	−3.9 (31.8)	5.5 (39.4)	NS*	14.6 (38.3)	0.6 (32.6)	−3.5 (39.1)	−8.7 (30.9)
Lp (a)	5.0 (28.6)	9.4 (29.6)	NS*	6.9 (25.3)	7.9 (33.0)	NS*	−2.1 (27.3)	11.8 (29.0)	16.9 (35.7)	2.6 (21.3)
Weight/BMI	−0.1 (2.4)	−0.9 (2.9)	NS	−0.1 (2.8)	−0.8 (2.4)	NS	−0.8 (2.0)	0.6 (2.6)	−0.9 (2.9)	−0.9 (2.9)

P-values are from the general linear model. In case of a non-parametric distribution, the van-Elteren test was used (indicated by *). In the upper part changes from baseline to 4 weeks, in the lower part from baseline to 20 weeks.

## Discussion

Our study demonstrates that in patients with carotid arteriosclerosis, both, LC and red wine have a beneficial effect on the LDL/HDL ratio after 20 weeks with an 8% and 13% decrease, respectively when compared to their control groups. This effect is already present after 4 weeks in the red-wine intervention group. LDL is significantly lowered in the LC group. HDL increased after 4 weeks in the red-wine group. HDL was hardly affected by LC. The effect of red wine on LDL/HDL was independent from the lifestyle changes, i.e. there was an additional benefit. This is a remarkable result as the majority of our patients (65%) were already on statin treatment and baseline LDL was already low (104.9 mg/dl). Therefore both, healthy diet and increased physical activity on the one hand and a glass of red wine (0.2 l/day for men and 0.1 l/day for women) on the other hand improve the ratio of LDL and HDL in cerebrovascular patients. The effect on LDL/HDL ratio after 20 weeks was, however, more pronounced in the non-LC group. This is probably due to a ceiling effect. Our study is so far the largest prospective randomised study using red wine. Total cholesterol (−6%) and triglycerides (−13%) were lowered by LC when compared to the non-LC group after 20 weeks, not after 4 weeks. Lp(a) was not affected by any intervention.

The beneficial effect of Mediterranean diet on lipids is well documented. In their recent meta-analysis, Kastorini et al. found an increase in HDL of 1.17 mg/dl and a decrease in triglycerides of 6.14 mg/dl when comparing a Mediterranean diet with a conventional diet [14]. Some of the studies included alcohol/red wine, others not. Our study is so far the first to document an additional effect of adding red wine to a Mediterranean diet on blood lipids. Sola et al. investigated the effect of a Mediterranean diet enriched in mixed nuts in 193 high cardiovascular risk subjects after 3 months [13]. Compared with baseline, total cholesterol, LDL, the ratio LDL/HDL, and triglycerides decreased significantly by 3.1%, 4.2%, 4.8%, and 6.2%, respectively. HDL increased by 1.8%. Regular physical exercise also has a beneficial effect on lipids, HDL is increased by about 8%, triglycerides are reduced by about 27% [11]. LDL and total cholesterol are hardly affected. We included in our diet 3 items known to alter positively lipid profile and generally accepted in the European culture: dark chocolate, tomatoes and walnuts, the latter two being frequently part of a Mediterranean diet [15-20].

Previous studies have investigated the effect of LC and red wine on cholesterol. Avellone et al. investigated the effect of 250 ml of red wine daily on cholesterol in 48 subjects over 4 weeks. They found a 10% increase of HDL (significant) and a trend of a 7% decrease of LDL (not significant) with the intervention. LDL/HDL improved significantly by 14% [42]. This number of 14% is comparable to the reduction of LDL/HDL by 13% found in the present study when comparing the results of the wine group to the non-alcohol group. In their study, triglycerides decreased by 7%, and Lp(a) by 21%, however not significantly.

Coimbra et al. investigated 16 healthy adult subjects with isolated hypercholesterolemia who had to drink 500 ml of red grape juice or 250 ml of red wine daily. There was no effect on LDL, HDL, triglycerides and Lp(a) after 2 weeks (compared with baseline) [26]. The duration of the study may have been too short; however in another study over 2 weeks, Rifler et al. investigated the effect of 250 ml of red wine daily in 33 patients post myocardial infarction [27,43]. Both groups received a western prudent diet and performed adapted exercise and physiotherapy. After 2 weeks the wine drinkers had a 5% decrease in total cholesterol and LDL, respectively, as compared to baseline, but a 16% and 18% decrease in total cholesterol and LDL compared to the no-wine group. Triglycerides did not change. Although the data of the no-wine group compared to baseline are not given in the article, this means that the no-wine group showed an increase in LDL and total cholesterol, a finding also observed in our study. A possible explanation for this phenomenon is that in our study and in the study by Rifler et al. (in hospitalised patients) the no-wine group is as a matter of fact a no-alcohol or stop-alcohol group. Probably part of these subjects had consumed alcohol before the study and had to stop during the study.

In the literature there is conflicting evidence whether Lp(a) can be affected by lifestyle changes or not, like in our study. Lp(a) decreased after the regular daily ingestion of red wine with 30 g alcohol [44]. In 2 other studies Lp(a) increased after alcohol withdrawal [45,46]. In a postmenopausal women, no effect of two alcoholic drinks a day on Lp(a) was observed [47]. In a Norwegian study those who exercised increased their Lp(a) levels with 15.4 (S.E. = 8.0) mg/l as compared to no exercise (P < 0.05). Also, dietary intervention tended to increase Lp(a), but the increase did not reach statistical significance [48].

Our study did not address the intriguing question whether there is a particularly beneficial effect of red wine over other forms of alcohol concerning the effect on blood lipids. Recent reviews suggest that regular consumption of small quantities of any form of alcohol prevents cerebro-cardiovascular diseases rather than that there is a particular benefit of red wine [21,24,49].

Potential limitations of our study are that the study size was modest, this was an open trial, clinical benefit was not shown, and the intervention follow-up time was rather short (20 weeks only). The strength of the study is the demonstration of an additional beneficial effect of red wine on top of otherwise healthy lifestyle on blood lipids.

## Conclusions

Lifestyle changes including a modified Mediterranean diet and physical exercise as well as a glass of red wine daily improve independently from each other LDL/HDL ratio in patients with carotid arteriosclerosis even though the vast majority was already on statin therapy. This may also translate in a reduction of future heart attacks and strokes. The exact molecules and mechanisms of the beneficial effect of red wine on blood lipids can be deciphered in future studies.

## Abbreviations

BMI: Body mass index; HDL: High density lipoprotein; LC: Lifestyle changes; LDL: Low density lipoprotein; Lp (a): Lipoprotein (a).

## Competing interests

The authors declare that they have no competing interests.

## Authors’ contributions

DWD, CI, MV, MM, MG, TV, GG, and AC developed the study design and organized its performance; TV, DWD, MM, and GG performed the data collection; NDB, CL, and AK performed the data monitoring and administration; MV and TG performed the statistical analyses; DWD wrote the manuscript; all were involved in data interpretation and read and approved the final manuscript.

## References

[B1] Amarenco P Labreuche J Lipid management in the prevention of stroke: review and updated meta-analysis of statins for stroke prevention Lancet Neurol 2009 8 453 463 10.1016/S1474-4422(09)70058-4 19375663

[B2] Goldstein LB Bushnell CD Adams RJ Appel LJ Braun LT Chaturvedi S Guidelines for the primary prevention of stroke: a guideline for healthcare professionals from the American heart association/american stroke association Stroke 2011 42 517 584 10.1161/STR.0b013e3181fcb238 21127304

[B3] Taylor F Huffman MD Macedo AF Moore THM Burke M Davey Smith G Ward K Ebrahim S Statins for the primary prevention of cardiovascular disease Cochrane Database of Systematic Reviews 2013 Issue 1 Art. No.: CD004816 DOI: 10.1002/14651858.CD004816.pub5 10.1002/14651858.CD004816.pub5 PMC6481400 23440795

[B4] Genser B Dias KC Siekmeier R Stojakovic T Grammer T Maerz W Lipoprotein (a) and risk of cardiovascular disease–a systematic review and meta analysis of prospective studies Clin Lab 2011 57 143 156 21500721

[B5] Enkhmaa B Anuurad E Zhang W Tran T Berglund L Lipoprotein(a): genotype-phenotype relationship and impact on atherogenic risk Metab Syndr Relat Disord 2011 9 411 418 10.1089/met.2011.0026 21749171 PMC3225061

[B6] Muntner P Lee F Astor BC Association of high-density lipoprotein cholesterol with coronary heart disease risk across categories of low-density lipoprotein cholesterol: the atherosclerosis risk in communities study Am J Med Sci 2011 341 173 180 10.1097/MAJ.0b013e3181f97e4a 21042169

[B7] Huxley RR Barzi F Lam TH Czernichow S Fang X Welborn T Isolated Low levels of high-density lipoprotein cholesterol Are associated with an increased risk of coronary heart disease: an individual participant data meta-analysis of 23 studies in the asia-pacific region Circulation 2011 124 2056 2064 10.1161/CIRCULATIONAHA.111.028373 21986289

[B8] Demarin V Lisak M Morovic S Cengic T Low high-density lipoprotein cholesterol as the possible risk factor for stroke Acta Clin Croat 2010 49 429 439 21830454

[B9] Acharjee S Qin J Murphy SA McCabe C Cannon CP Distribution of traditional and novel risk factors and their relation to subsequent cardiovascular events in patients with acute coronary syndromes (from the PROVE IT-TIMI 22 trial) Am J Cardiol 2010 105 619 623 10.1016/j.amjcard.2009.10.042 20185006

[B10] Labreuche J Deplanque D Touboul PJ Bruckert E Amarenco P Association between change in plasma triglyceride levels and risk of stroke and carotid atherosclerosis: systematic review and meta-regression analysis Atherosclerosis 2010 212 9 15 10.1016/j.atherosclerosis.2010.02.011 20457452

[B11] Trejo-Gutierrez JF Fletcher G Impact of exercise on blood lipids and lipoproteins J Clin Lipidol 2007 1 175 181 10.1016/j.jacl.2007.05.006 21291678

[B12] Brown WV Fletcher GF Wilson PW Using exercise to reduce risk J Clin Lipidol 2009 3 360 367 10.1016/j.jacl.2009.10.006 21291837

[B13] Sola R Fito M Estruch R Salas-Salvado J Corella D de La TR Effect of a traditional mediterranean diet on apolipoproteins B, a-I, and their ratio: a randomized, controlled trial Atherosclerosis 2011 218 174 180 10.1016/j.atherosclerosis.2011.04.026 21640348

[B14] Kastorini CM Milionis HJ Esposito K Giugliano D Goudevenos JA Panagiotakos DB The effect of mediterranean diet on metabolic syndrome and its components: a meta-analysis of 50 studies and 534,906 individuals J Am Coll Cardiol 2011 57 1299 1313 10.1016/j.jacc.2010.09.073 21392646

[B15] Buitrago-Lopez A Sanderson J Johnson L Warnakula S Wood A Di AE Chocolate consumption and cardiometabolic disorders: systematic review and meta-analysis BMJ 2011 343 d4488 10.1136/bmj.d4488 21875885 PMC3163382

[B16] Tokede OA Gaziano JM Djousse L Effects of cocoa products/dark chocolate on serum lipids: a meta-analysis Eur J Clin Nutr 2011 65 879 886 10.1038/ejcn.2011.64 21559039

[B17] Blum A Merei M Karem A Blum N Ben Arzi S Wirsansky I Effects of tomatoes on the lipid profile Clin Invest Med 2006 29 298 300 17144439

[B18] Silaste ML Alfthan G Aro A Kesaniemi YA Horkko S Tomato juice decreases LDL cholesterol levels and increases LDL resistance to oxidation Br J Nutr 2007 98 1251 1258 17617941 10.1017/S0007114507787445

[B19] Sabate J Oda K Ros E Nut consumption and blood lipid levels: a pooled analysis of 25 intervention trials Arch Intern Med 2010 170 821 827 10.1001/archinternmed.2010.79 20458092

[B20] Banel DK Hu FB Effects of walnut consumption on blood lipids and other cardiovascular risk factors: a meta-analysis and systematic review Am J Clin Nutr 2009 90 56 63 10.3945/ajcn.2009.27457 19458020 PMC2696995

[B21] Krenz M Korthuis RJ Moderate ethanol ingestion and cardiovascular protection: from epidemiologic associations to cellular mechanisms J Mol Cell Cardiol 2012 52 93 104 10.1016/j.yjmcc.2011.10.011 22041278 PMC3246046

[B22] Smoliga JM Baur JA Hausenblas HA Resveratrol and health–a comprehensive review of human clinical trials Mol Nutr Food Res 2011 55 1129 1141 10.1002/mnfr.201100143 21688389

[B23] Kloner RA Rezkalla SH To drink or not to drink? that is the question Circulation 2007 116 1306 1317 10.1161/CIRCULATIONAHA.106.678375 17846344

[B24] O’Keefe JH Bybee KA Lavie CJ Alcohol and cardiovascular health: the razor-sharp double-edged sword J Am Coll Cardiol 2007 50 1009 1014 10.1016/j.jacc.2007.04.089 17825708

[B25] Avellone G Di GV Campisi D De Simone R Raneli G Scaglione R Effects of moderate Sicilian red wine consumption on inflammatory biomarkers of atherosclerosis Eur J Clin Nutr 2006 60 41 47 10.1038/sj.ejcn.1602265 16132058

[B26] Coimbra SR Lage SH Brandizzi L Yoshida V da Luz PL The action of red wine and purple grape juice on vascular reactivity is independent of plasma lipids in hypercholesterolemic patients Braz J Med Biol Res 2005 38 1339 1347 10.1590/S0100-879X2005000900008 16138217

[B27] Flensborg-Madsen T Knop J Mortensen EL Becker U Makhija N Sher L Beverage preference and risk of alcohol-use disorders: a Danish prospective cohort study J Stud Alcohol Drugs 2008 69 371 377 18432379 10.15288/jsad.2008.69.371

[B28] Estruch R Ros E Salas-Salvado J Covas MI Pharm D Corella D Primary prevention of cardiovascular disease with a mediterranean diet N Engl J Med 2013 368 1279 1290 10.1056/NEJMoa1200303 29897867

[B29] Touboul PJ Hennerici MG Meairs S Adams H Amarenco P Bornstein N Mannheim carotid intima-media thickness and plaque consensus (2004-2006-2011). An update on behalf of the advisory Board of the 3rd, 4th and 5th watching the risk symposia, at the 13th, 15th and 20th European Stroke Conferences, Mannheim, Germany, 2004, Brussels, Belgium, 2006, and Hamburg, Germany, 2011 Cerebrovasc Dis 2012 34 290 296 10.1159/000343145 23128470 PMC3760791

[B30] Arning C Widder B von Reutern GM Stiegler H Gortler M [Revision of DEGUM ultrasound criteria for grading internal carotid artery stenoses and transfer to NASCET measurement] Ultraschall Med 2010 31 251 257 10.1055/s-0029-1245336 20414854

[B31] von Reutern GM Goertler MW Bornstein NM Del SM Evans DH Hetzel A Grading carotid stenosis using ultrasonic methods Stroke 2012 43 916 921 10.1161/STROKEAHA.111.636084 22343647

[B32] Droste DW Keipes M The reduction of stroke risk, risk of myocardial infarction and death by healthy diet and physical activity Bull Soc Sci Med Grand Duche Luxembin press 24437075

[B33] Mente A de Koning L Shannon HS Anand SS A systematic review of the evidence supporting a causal link between dietary factors and coronary heart disease Arch Intern Med 2009 169 659 669 10.1001/archinternmed.2009.38 19364995

[B34] Ministère de la Santé Le plaisir de bien manger et d'être actif! 2011 Luxembourg http://www.sante.public.lu/publications/rester-bonne-sante/alimentation/plaisir-bienmanger-etre-actif/plaisir-bien-manger-etre-actif-2011-de.pdf

[B35] Powell KE Paluch AE Blair SN Physical activity for health: what kind? How much? How intense? on top of what? Annu Rev Public Health 2011 32 349 365 10.1146/annurev-publhealth-031210-101151 21128761

[B36] Damasceno NR Perez-Heras A Serra M Cofan M Sala-Vila A Salas-Salvado J Crossover study of diets enriched with virgin olive oil, walnuts or almonds. Effects on lipids and other cardiovascular risk markers Nutr Metab Cardiovasc Dis 2011 21 1 S14 S20 21421296 10.1016/j.numecd.2010.12.006

[B37] Mursu J Voutilainen S Nurmi T Rissanen TH Virtanen JK Kaikkonen J Dark chocolate consumption increases HDL cholesterol concentration and chocolate fatty acids may inhibit lipid peroxidation in healthy humans Free Radic Biol Med 2004 37 1351 1359 10.1016/j.freeradbiomed.2004.06.002 15454274

[B38] Elkind MS Sciacca R Boden-Albala B Rundek T Paik MC Sacco RL Moderate alcohol consumption reduces risk of ischemic stroke: the Northern Manhattan study Stroke 2006 37 13 19 10.1161/01.STR.0000195048.86810.5b 16306464

[B39] Wilkins K Moderate alcohol consumption and heart disease Health Rep 2002 14 9 24 15069799

[B40] Pereira MA Swain J Goldfine AB Rifai N Ludwig DS Effects of a low-glycemic load diet on resting energy expenditure and heart disease risk factors during weight loss JAMA 2004 292 2482 2490 10.1001/jama.292.20.2482 15562127

[B41] Singh RB Rastogi SS Niaz MA Ghosh S Singh R Gupta S Effect of fat-modified and fruit- and vegetable-enriched diets on blood lipids in the Indian Diet Heart Study Am J Cardiol 1992 70 869 874 10.1016/0002-9149(92)90729-I 1529939

[B42] Avellone G Di GV Campisi D Alonzo G Gambino L Avellone G [Effects of two Sicilian red wines on some cardiovascular risk factors] Ital Heart J Suppl 2004 5 382 388 15182065

[B43] Rifler JP Lorcerie F Durand P Delmas D Ragot K Limagne E A moderate red wine intake improves blood lipid parameters and erythrocytes membrane fluidity in post myocardial infarct patients Mol Nutr Food Res 2011 56 345 351 22419533 10.1002/mnfr.760

[B44] Chiva-Blanch G Urpi-Sarda M Ros E Valderas-Martinez P Casas R Arranz S Effects of red wine polyphenols and alcohol on glucose metabolism and the lipid profile: a randomized clinical trial Clin Nutr 2012 32 200 206 22999066 10.1016/j.clnu.2012.08.022

[B45] Budzynski J Klopocka M Swiatkowski M Pulkowski G Ziolkowski M Lipoprotein(a) in alcohol-dependent male patients during a six-month abstinence period Alcohol Alcohol 2003 38 157 162 10.1093/alcalc/agg045 12634264

[B46] Kervinen K Savolainen MJ Kesaniemi YA A rapid increase in lipoprotein (a) levels after ethanol withdrawal in alcoholic men Life Sci 1991 48 2183 2188 10.1016/0024-3205(91)90152-2 1827857

[B47] Clevidence BA Reichman ME Judd JT Muesing RA Schatzkin A Schaefer EJ Effects of alcohol consumption on lipoproteins of premenopausal women. A controlled diet study Arterioscler Thromb Vasc Biol 1995 15 179 184 10.1161/01.ATV.15.2.179 7749823

[B48] Holme I Urdal P Anderssen S Hjermann I Exercise-induced increase in lipoprotein (a) Atherosclerosis 1996 122 97 104 10.1016/0021-9150(95)05761-7 8724116

[B49] Ronksley PE Brien SE Turner BJ Mukamal KJ Ghali WA Association of alcohol consumption with selected cardiovascular disease outcomes: a systematic review and meta-analysis BMJ 2011 342 d671 10.1136/bmj.d671 21343207 PMC3043109

